# Essentials in Dental Education

**Published:** 1904-12

**Authors:** 


### Editorial.

### ESSENTIALS IN DENTAL EDUCATION.

What is to be understood by this use of words? Does this mental training in any sense differ from any other education in the final results? Is not all education, however effected, but a cultivation of the brain-cells and, in addition thereto, reflex power, represented in thought? These are questions of great importance, yet we are met with the assertion that education, to be of value, must be acquired through certain methods of training, an inheritance from mediæval periods, and that professional culture has no place in the mental standards of the twentieth century.

To the writer this view is radically wrong and unworthy the great accomplishments of this utilitarian age.

It is a problem remaining unsettled with many, whether it is possible to make a thinker—a philosopher—unless the education is based exclusively upon what are known as the humanities. This is the natural outcome of the standard of learning established centuries ago, and may find its best example in the study given to Confucius and his works by the recognized scholars of China. It is not the purpose, however, of this article to discuss this much-mooted question, and it is only alluded to here as a basis for related thought.

Dental education as understood fifty years ago was simply the acquirement of a trade. It consisted in its last analysis in the manufacture of artificial masticating substitutes and the stopping or filling cavities in teeth to prevent further destruction by caries. The man who labored in the laboratory found his work a succession of repetitions, doing to-morrow what he did the day previously, and the man over the chair had no other expectations or higher ambitions, each branch aiming at greater perfection as experience necessarily gave manipulative skill. There was but a limited sphere here for thought. The tendency was to a life of repetitions, and repetitions had the usual result of narrow thinking. There was no room for the broader life common to the older professions. The work was regarded as useful and necessary, but the men who followed it were held not to be worthy the consideration due professional men, and their claim to be such was derided, and socially they became, in a certain sense, outcasts and their claims to the doctor title was regarded as an assumption.

The fathers in dentistry were not satisfied with this anomalous position, and the effort was made to increase the curriculum to something more nearly that of medicine. The slow development of this through over sixty years has been fruitful through its slow progress in the evolution of acquirement that at this period marks it as equal to the mother profession in broader conceptions and more accurate modes of thinking and practice. The man in dentistry to-day aspires to a mental power that will enable him to hold communion with the more profound thinkers of his own and past ages. He is no longer satisfied with the nomenclature of his profession. He has cast aside the word mechanics, as related to artificial substitutes, and now labors under the prosthetic name. He is beginning to feel that the word dentist does not comport with the dignity of his calling, and recognizes that stomatology more nearly answers to his higher thought. The man who regulated teeth in the earlier periods is now an orthodontist, and the continuous-gum worker has been transformed into a practitioner in ceramics. This is encouraging. The superficial thinker may laugh and regard it as bordering on shallow conceit, but to the thoughtful mind it gives a positive evidence of a growing taste for the artistic in thought as well as in practice, for its indications, while limited, point unerringly to a higher standard through the evolution of ideas.

The curriculum of forty years ago, in the few dental colleges then existing, consisted of operative dentistry, didactic and practical; mechanical dentistry the same; a minimum of anatomy through lectures; lectures on chemistry with no practice in the laboratory; materia medica from the stand-point of the medical practitioner; the therapeutics limited to few drugs, and those used empirically; dental pathology was scarcely recognized as a distinct study, and, above all, there was no standard of entrance. To-day the diploma of a four years' high school, or its equivalent, is demanded by the higher schools. This, as a preliminary, presupposes higher thinking and a dissatisfaction with the drudgery regarded as essential in the older methods of work. The man of to-day is finding his place, not so much in what is called the mere mechanics of his profession, but in the elaborating of its more artistic features that have in the past been mainly neglected.

He is brought in contact in his first year with the sculptor, modeller. He must rise to the conception of the beauty of form of the human mouth and all that it contains. He must think as he works over his chemical problems in the laboratory. He must grasp the limitations of his human intellect as he searches for the ultimate in his histological work. He will gather new inspiration as he studies the life history of the minute forms of activity in his bacteriological examinations. He will over the cadaver speculate on the dynamic power of the muscles, activity of the nerves, and the strength of the frame, and he must watch the knife of the surgeon as he skilfully dissects out the tumor or calls to his aid the mechanism prepared by the stomatologist to assist nature while she restores normal powers to the fractured jaw. He listens to lectures and works, it may be, in the physiological and pathological laboratory,—in a word, he has become in the evolution of sixty years a thinker and a professional man.

This may not be true of all the dental colleges of the country, but it will be true in the not far distant future. The higher ideals are being grasped and slowly but surely are being made part of the curriculi of the schools, and before the curtain is rung down on the present century the thorough training outlined will be regarded as the essentials in dental education.

This brings the thoughtful individual to the consideration of the question, Have we, as dentists, lost or gained in this gradual evolution? It must be evident that there has been both a loss and a gain. We have lost to a large extent prosthetic ability. We have also lost in our manipulative skill in operative dentistry. This, we are aware, will be denied; but taking the country through, the percentage of skilled operators will not compare with those of thirty or forty years in the past. In a word, then, the mechanical side of dentistry is gradually giving way to a higher professional culture, and this, in the progressive development of time, must be relegated to a special class. It is difficult to view this possible change with any other feeling than that of profound regret. Prosthetic dentistry is, in the view of the writer, the second greatest boon given to humanity during the nineteenth century. It ranks, in his opinion, a close second to the discovery of anæsthesia. It ought not to be cast aside as unworthy the highest efforts of the thinker; indeed, it should be to him, more than aught else, the very foundation and support of that higher spiritual life manifest in the human brain. It has extended the life of the race, but while this is important, it has not the same value as is its service in keeping the digestive apparatus in order and through this normal activity holding the nerve forces in harmonious and in working condition, thus preserving mentality to the last days of life. If there is anything higher than this in any profession the writer is not aware of its existence.

The art of filling teeth as understood fifty years ago and at present will undoubtedly give place to something better, at least as far as material is concerned. This is not to be mourned. The dentist has been too long the slave of a master of his own creating, and the release from the drudgery will be a happy fulfilment, when it comes, of the hopes of many years.

The bright side of this picture is the release it will give the practitioner and a larger devotion to those studies that make for a more liberal view of life and its duties, and those things that count for the spiritual elevation of man. To-day the dental practitioner is a drudge, not the drudge of fifty years ago, but still bowed down over his daily work for daily bread. He fails to grasp the possibilities lying dormant in his profession. He reads but little and thinks less. His days are given over to labor and his nights to disturbed repose. He has no hours and no desire to devote to the cultivation of those amenities that go so far to make beautiful our civilization. It is time he was aroused from this lethargic state, and the question of the hour is, How may this be accomplished? Education, as already stated, has done much, will do more; but we need an education that will lead the dentists of the period out of the morass of mere money-getting into the more perfect soulful life of the thinker.

Are we viewing dental education from the stand-point of the real educators in dentistry? Are we not laying down uncertain laws that will land our profession again into the depths from which we have laboriously emerged? There seems to be in a certain class of mind a feeling of rejoicing that the dental colleges of this country halted progress in this direction and turned the wheels of our civilization backward. When that body assuming the name of the National Association of Dental Faculties met at St. Louis and adopted three years as the course of college training, it cared nothing for higher education. The slogan was sounded broadcast, "We will be financially ruined if this four years' course is continued." Gold, gold, gold, has been the cry of the besotted world in all ages, drowning the "still small voice crying in the wilderness" of confused thought. Come out of the temples dedicated to the brazen god and in the clearer atmosphere of a broader culture breathe a new inspiration and a more perfect life.

Recently one of our best writers, in discussing this subject of dental education in colleges, cried, in tones of agony, "Time! Time! Time! Always time, as though the length of a term was a true measure of the quality of the education imparted." Yes. Time we must have. There can be no real value in hurried work. The brain-cells need time for an impression. The superficial mark fades rapidly. There is such a thing as indigestion of the brain-forces. They become over-saturated and refuse to further act. The power to think is measurably lost. That some may be able to accomplish in two years that which requires four in others is no argument to prove that the two years' man will be the standard bearer in the future; indeed, in the experience of the writer this has been reversed in almost every instance. The man of two years has taken his intellectual pabulum too rapidly, with the result—mental indigestion.

The idea entertained that three years of nine months each is equal to four years of seven months is based on wrong conclusions and imperfect knowledge of the possibilities of dental education. Every one practically familiar with dental training is aware that the last two months of a nine months' course are the least profitable months, and relatively so of a shorter period. The human mind staggers under the continuous load and must have rest and time for recovery. For this the fourth year is imperatively necessary.

Some of the dental colleges tried bravely to resist the current that threatened to engulf them. That they failed is now an historical record, but their day of triumph will surely come. This will be when dentists cease to worship mammon. When they cling closely to their higher conceptions, treading under feet all baser motives. When they give their influence for a dental education that will draw out all that there is in a man. When they cultivate the best in literature and cast away that which drags down the profession. In a word, when they aim for the highest attainable.

The time may come when the man who studies will be graded according to his ambitions. The prosthetic worker may conclude his preparation in three years, the operative expert in four, and the stomatologist in five. Whether this will be the logical outcome of educational effort remains to be seen, but it is to be hoped that whatever course may be adopted it will be to the honor of dentistry and the broadening of the mental horizon of all who labor within its folds.

